# Computed Tomography and Magnetic Resonance Imaging Findings of a Malignant Hepatic Epithelioid Hemangioendothelioma: A Rare Case of Solitary Small Nodular Form

**DOI:** 10.1177/2324709613504549

**Published:** 2013-09-13

**Authors:** Soo-heui Baek, Jung-Hee Yoon

**Affiliations:** 1 Inje University Haeundae Paik Hospital, Busan, Republic of Korea

**Keywords:** gadoxetic acid (Gd-EOB-DTPA)–enhanced MRI, liver, capsular retraction, neoplasms, epithelioid hemangioendothelioma

## Abstract

We report the case of a rare solitary small nodular form of malignant hepatic epithelioid hemangioendothelioma in a patient followed by computed tomography and gadoxetic acid (Gd-EOB-DTPA)–enhanced magnetic resonance imaging with histological analysis. This case showed early peripheral septal and nodular enhancement and delayed centripetal enhancing pattern with capsular retraction, mimicking peripheral cholangiocarcinoma, inflammatory pseudotumor, or metastases. The histological and immunohistochemical findings were diagnostic of a malignant hepatic epithelioid hemangioendothelioma.

## Introduction

Epithelioid hemangioendothelioma (EHE) is a rare, low- to intermediate-grade malignant vascular tumor that originates from the soft tissue, visceral organs, bone, lung, brain, and small intestines.^1^ It was first defined by Weiss and Enzinger^2^ in 1982 as a soft tissue vascular tumor of endothelial origin with a clinical course between that of benign hemangioma and angiosarcoma. Most hepatic EHEs present as the diffuse multifocal type, which is an advanced stage, and rarely as the nodular type, which represents an early stage^3,4^ and more commonly affects adult females with a peak incidence between 30 and 50 years of age.^4^ Treatment options include liver resection or liver transplantation, and the prognosis is more favorable than that of other hepatic malignancies.^4^ Metastases have been reported in 27% to 37% of patients at presentation and occur most commonly in the lungs.

Generally, a specific diagnosis for the nodular type is difficult without a biopsy because the radiologic findings are similar to those for some hepatic metastases. The diffuse form of EHE has more specific diagnostic criteria, and peripheral location and capsular retraction are hallmarks of hepatic EHE.

We present a case of a rare solitary small nodular form of malignant hepatic EHE.

## Case

**Figure 1.**
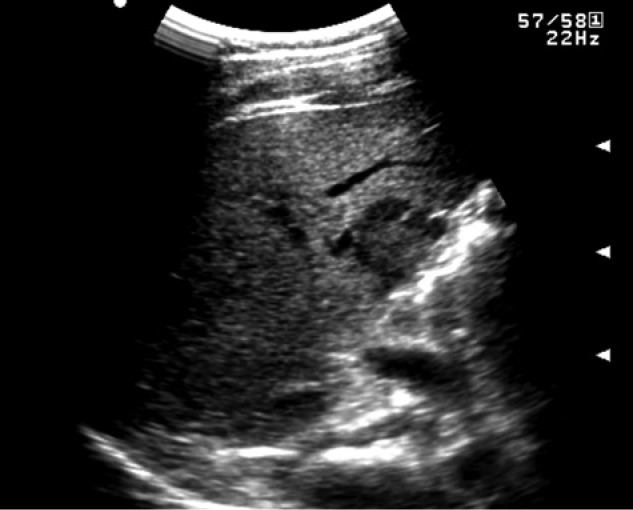
Abdominal ultrasonography image of a 22-year-old man without underlying liver disease shows a well-defined, inhomogeneous echoic hepatic nodule with a predominantly hypoechoic rim in the periphery of the right hepatic lobe.

**Figure 2.**
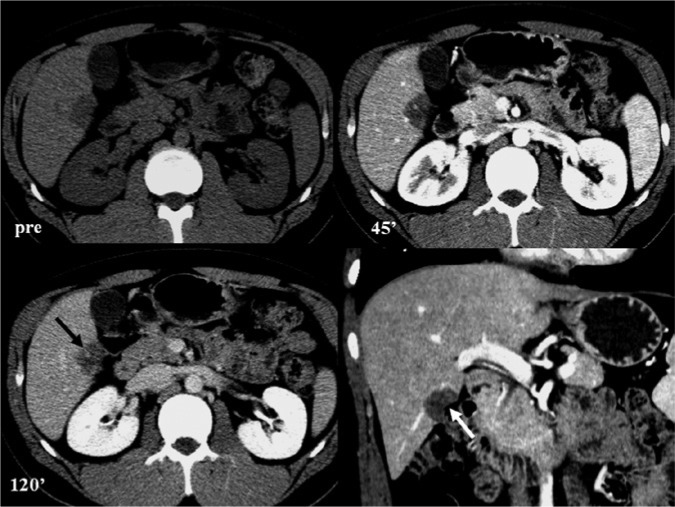
Multidetector computed tommography scan axial and coronal images. Precontrast axial image (upper left) shows the following: well-defined, low attenuating mass to the normal liver parenchyma. Arterial (upper right)/equilibrium (lower left) axial image shows the following: the lesion was suspicious for peripheral enhancement (black arrow). Coronal reconstruction of arterial phase (lower right) shows the following: capsular retraction was well delineated (white arrow).

**Figure 3.**
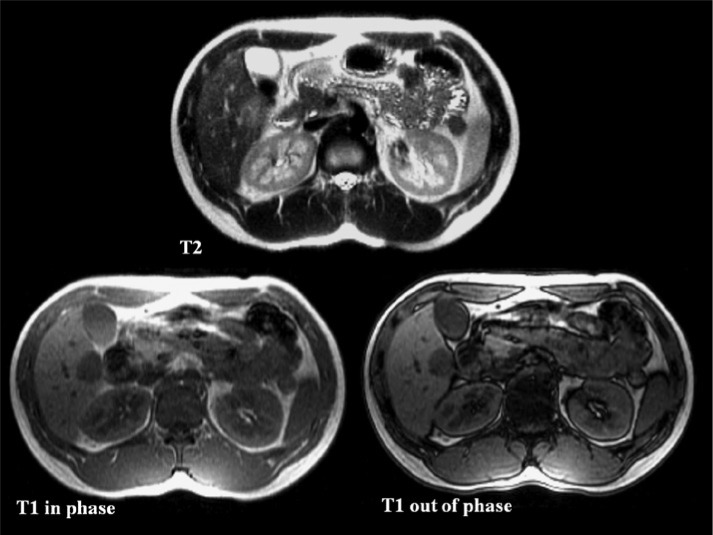
Gadoxetic acid (Gd-EOB-DTPA)–enhanced magnetic resonance images. T2-weighted axial imaging (upper) shows the following: well-circumscribed hepatic nodule, central very high signal intensity with intermediate high signal in the periphery. T1-weighted in-phase (lower left)/out-of- phase (lower right) axial shows the following: low signal intensity without signal drop of fat.

**Figure 4.**
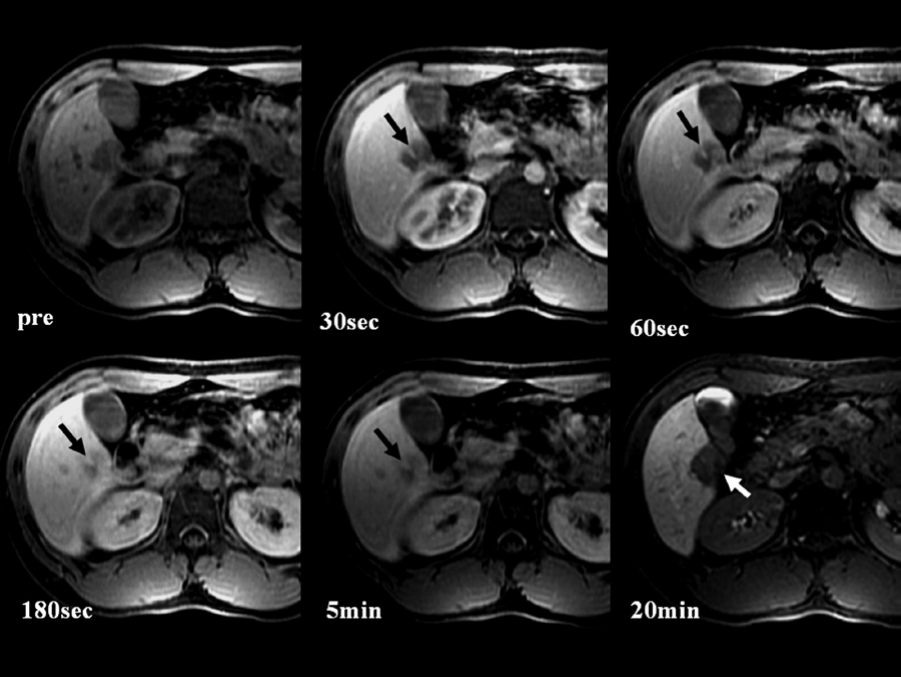
Gadoxetic acid (Gd-EOB-DTPA)–enhanced magnetic resonance images, contrast-enhanced T1-weighed image shows the following: low signal intensity hepatic nodule on the precontrast T1-weighted image (upper left), showing peripheral septa-like enhancement (black arrow) in the arterial phase (upper middle), and more globular centripetal enhancement during the portal venous phase (upper right), the equilibrium phase (lower left) to 5 minutes delayed phase (lower middle), and became low signal intensity on the hepatobiliary phase (lower right) with focal capsular retraction (white arrow).

**Figure 5.**
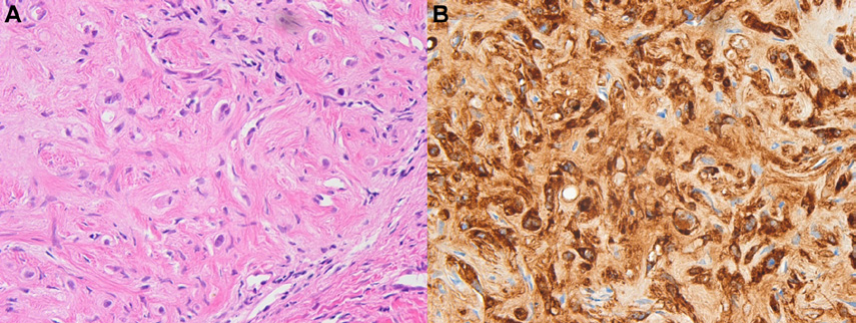
Microscopic and immunohistochemical findings of hepatic epithelioid hemangioendothelioma. Photomicrograph shows epithelioid differentiation and abundant intracytoplasm with signet ring-like structure (A, hematoxylin and eosin stain; original magnification, 400×), immune staining for endothelial markers (factor VIII–associated antigen) show positive tumor cells (B).

A 22-year-old man without underlying liver disease or clinical symptom visited our hospital for screening. The patient was negative for hepatitis B surface antigen and hepatitis C virus antibody, and the serum α-fetoprotein level was within normal limits. On ultrasonography, a 2.3 cm incidental hepatic lesion was detected that was a well-defined, inhomogeneous hypoechoic hepatic nodule that extended to the hepatic surface (Figure 1). Multidetector computed tomography (CT) was performed to characterize the focal liver lesion using 100 mL of a nonionic contrast medium (Omnipaque 350, GE Healthcare, Waukesha, WI), at a rate of 3 mL/s. This hepatic nodule was well defined with low attenuation at the periphery of the right lobar segment V and peripheral enhancement during the arterial and delayed phase (Figure 2). A definite focal capsular retraction was found adjacent to the nodule, which was better delineated on the coronal reconstruction image (Figure 2). Magnetic resonance (MR) imaging was obtained with a 1.5-T unit using a liver-specific contrast agent, gadoxetic acid (Gd-EOB-DTPA, Primovist, Bayer Schering Pharma AG, Berlin, Germany). On T2-weighted MR imaging (TR/TE = 1571.2/88.2), the nodule showed very high signal intensity (SI) on the center with intermediate high SI on the periphery and low SI on T1-weighted in-phase MR imaging (TR/TE = 150.0/2.2; Figure 3) without signal loss on out-of-phase images (TR/TE = 100/1.9; Figure 3). On gadoxetic acid–enhanced MR imaging, the mass demonstrated peripheral septal or nodular enhancement during the early arterial phase and more globular centripetal enhancement during the portal venous and equilibrium phase and showed a low SI defect with an area of capsular retraction in the hepatobiliary phase (Figure 4). We believe that peripheral septal or nodular enhancement is suggestive of the vascular architecture; therefore, these enhancing patterns and capsular retractions were key imaging features in this case. We think that these incidental hepatic nodules may represent inflammatory pseudotumor, metastases, or peripheral cholangiocarcinoma. Biopsy was performed with ultrasound guidance, and histology suggested a high-grade malignant neoplasm that originated from an endothelial cell. Based on these pathology results, a right sectionectomy was performed. Microscopic examination revealed that the neoplasm showed epithelioid differentiation and had an abundant intracytoplasm with cellular atypia and necrosis (Figure 5A).

Immunohistochemically, the tumor was positive for CD34 and factor VIII–related antigen, which are endothelial markers (Figure 5B). High-cell proliferation activity (MIB-1 expression index 10% to 30%) and overexpression of p53 by more than 10% were reported, which were suggestive indicators of biological aggressiveness. These microscopic and immunohistochemical findings were compatible with a high-grade malignant hepatic EHE.

Postoperative follow-up CT, bone scan, and ^18^F-FDG-PET/CT scan showed no evidence of distant metastasis or other primary tumors within 3 years. Therefore, we concluded that the final diagnosis was a primary malignant hepatic EHE.

## Discussion

Although most intrahepatic tumors are commonly associated with mass effects that result in contour bulging, 2.0% to 2.8% of tumors may show capsular retraction.^5,6^ Hepatic capsular retraction is a rare but specific sign in a variety of tumors, including EHE, liver metastases, cholangiocarcinomas, and fibrolamellar hepatocellular carcinoma.^6^

Liver metastases are the most common hepatic malignancies, and capsular retraction usually occurs in the subcapsular area, which is associated with systemic chemotherapy.^6^ In untreated hepatic metastases, capsular retraction can occur in the fibrosis-containing primary tumors such as colon cancer, breast cancer, carcinoid tumor, lung cancer, and gallbladder cancer.

Peripheral cholangiocarcinoma may cause capsular retraction because of an abundant fibrous stroma and chronic peripheral biliary obstruction. Therefore, if the hepatic tumor shows capsular retraction, delayed enhancement, and peripheral biliary dilatation, cholangiocarcinoma must be considered.^7^ However, in our case, biliary dilatation was not identified.

Hepatic EHE is a low- to intermediate-grade malignant vascular tumor, and it is important to differentiate hepatic EHE from other malignant tumors because long-term survival is possible after successful liver resection or liver transplantation.

Epithelioid hemangioendothelioma should not be confused with an infantile EHE, which is histologically benign, common in infants and young children, and resolves spontaneously.^8^

Most patients are asymptomatic when the tumor is detected and present with multifocal peripheral tumors. The solitary nodular form has been reported in only 13% to 18% of patients.^4^ The adjacent liver parenchyma is normal in the solitary small nodular form, whereas in the diffuse form, there is compensatory hepatic parenchymal enlargement and development of cirrhosis. Histologically, these tumors are composed of central fibrous myxoid stroma and peripheral rich cellularity with active proliferation of epithelioid and dendritic cells.^1,3^

Intravascular growth with neoplastic endothelial cell invasion results in fibrosis, progressive sclerosis, and calcification. The final diagnosis of EHE requires histopathological confirmation with immunohistochemical staining for endothelial markers. The vascular nature of the endothelial origin of the neoplastic cell is confirmed by positive factor VIII–related antigen staining and other endothelial cell markers (CD31, CD34).

The typical imaging findings are usually based on these histopathologic characteristics. Pathologically, hepatic EHE occurs mainly in the solitary type at the early stage, but with a propensity for invasion of terminal hepatic venules and portal vein branches.^9^ As a solitary hepatic EHE progresses, the tumor nodules become multifocal and confluent, especially in the subcapsular regions. Hepatic EHE shows intense peripheral enhancement due to active proliferation of epithelioid cells and a delayed or nonenhancing center due to dense fibrous stroma (target appearance). As they progress, fibrous septa within the tumor cause capsular retraction.^2^ The “capsular retraction” sign is caused by the fibroproliferative reactions of the tumors, which leads to invagination of the nearby liver capsule.^7,10,11^

Unenhanced CT imaging of a hepatic EHE is solid, with heterogeneously lower attenuation than normal liver parenchyma. After contrast enhancement, the tumor displayed no or faint peripheral rim enhancement at the arterial phase and a “halo” sign during the portal venous phase.^3^ The MR imaging of hepatic EHE includes heterogeneous intermediate to high SI on T2-weighted images and low SI on T1-weighted images. Heterogeneity on T2-weighted images may correspond to low signal zones of fibrotic, necrotic, or hemorrhagic areas, and higher signal zones of vascular channels and edematous connective tissues. On dynamic contrast-enhanced MR images, peripheral rim enhancement was better delineated. Gadoxetic acid is a hepatocyte-specific MR contrast agent that is increasingly used for liver MR imaging.

However, these findings are relatively nonspecific. According to Mehrabi et al,^4^ gadolinium-contrast enhanced MR imaging findings of 16 patients with hepatic EHE resulted in high enhancement (37.5%), peripheral and delayed central enhancement (37.5%), concentric layers of variable intensity (19%), and no enhancement (6%). In our case, the lesion was partially surrounded by a nonenhancing hypointense thin rim outside of the thick peripheral rim enhancement.

Miller et al^12^ reported that this avascular outer rim is caused from tumor invasion of hepatic sinusoids, venules, or small portal vein branches.

These previously reported characteristic CT and MR findings of hepatic EHE are in agreement with those of our case. Moreover, the hepatic nodule in our case has a capsular retraction with subcapsular location. Hepatic capsular retraction adjacent to a hepatic tumor was first described in the hepatic EHE, which reflects lesion-related fibrosis. Mehrabi et al^4^ reported marked capsular retraction in 10.6% of 142 patients with hepatic EHE. We initially thought that our patient had inflammatory pseudotumor. Inflammatory pseudotumors often have low attenuation on unenhanced images, low SI on T1-weighted images, and high SI on T2-weighted images with peripheral enhancement or enhancement of multiple internal septa.^13^ These imaging features correspond to those of our case. However, capsular retraction adjacent to hepatic nodules is rarely reported in inflammatory pseudotumor.^14^ When compared with the diffuse type of EHE, which reflects advanced-stage disease, the solitary nodular type, which is considered to be the earlier form of EHE, is often misdiagnosed because of its extreme rarity.^3,4^ Therefore, the final diagnosis of hepatic EHE requires histopathologic confirmation with immunologic staining for the endothelial markers.

However, based on this case, we concluded that the peripheral location of the tumor, capsular retraction, and peripheral enhancement are hallmark features. Certain clinical features such as the young age of patient and the good clinical condition as well as the positive imaging findings are suggestive of EHE.

Therefore, radiologists should be aware of the imaging findings associated with hepatic EHE and be able to suggest immunologic staining for endothelial markers for accurate diagnosis.
